# Predicting the Current and Future Potential Distributions of Lymphatic Filariasis in Africa Using Maximum Entropy Ecological Niche Modelling

**DOI:** 10.1371/journal.pone.0032202

**Published:** 2012-02-16

**Authors:** Hannah Slater, Edwin Michael

**Affiliations:** Department of Infectious Disease Epidemiology, Imperial College London, London, United Kingdom; University of Liverpool, United Kingdom

## Abstract

Modelling the spatial distributions of human parasite species is crucial to understanding the environmental determinants of infection as well as for guiding the planning of control programmes. Here, we use ecological niche modelling to map the current potential distribution of the macroparasitic disease, lymphatic filariasis (LF), in Africa, and to estimate how future changes in climate and population could affect its spread and burden across the continent. We used 508 community-specific infection presence data collated from the published literature in conjunction with five predictive environmental/climatic and demographic variables, and a maximum entropy niche modelling method to construct the first ecological niche maps describing potential distribution and burden of LF in Africa. We also ran the best-fit model against climate projections made by the HADCM3 and CCCMA models for 2050 under A2a and B2a scenarios to simulate the likely distribution of LF under future climate and population changes. We predict a broad geographic distribution of LF in Africa extending from the west to the east across the middle region of the continent, with high probabilities of occurrence in the Western Africa compared to large areas of medium probability interspersed with smaller areas of high probability in Central and Eastern Africa and in Madagascar. We uncovered complex relationships between predictor ecological niche variables and the probability of LF occurrence. We show for the first time that predicted climate change and population growth will expand both the range and risk of LF infection (and ultimately disease) in an endemic region. We estimate that populations at risk to LF may range from 543 and 804 million currently, and that this could rise to between 1.65 to 1.86 billion in the future depending on the climate scenario used and thresholds applied to signify infection presence.

## Introduction

The role of risk mapping in describing the spatial patterns of infection and guiding the planning of parasite control is now well-established, and has been demonstrated for a range of major parasitic diseases, including malaria [1], [2], trypanosomiasis [3], [4], schistosomiasis [5], [6], onchocerciasis [7], and lymphatic filariasis [8], [9], [10]. It has also led to an increased understanding of the climatic and environmental ecology of parasitic infections [8], [11], including improving appreciation of species thermal tolerances and the impact of key environmental variables on ecological traits that affect transmission, such as parasite development and survival rates. More recently, focus in parasite distribution modeling has expanded to evaluating the potential for the establishment and spread of invasive vector species [12], [13], [14] and assessing parasite or vector species responses to global climate change [15].

Lymphatic filariasis (LF) is a vector-borne infectious disease endemic in the tropics, including sub-Saharan Africa, and is thought to present the second largest public health burden of any disease worldwide [16]. The disease is transmitted to humans by infective mosquitoes that release parasitic filarial worms into the blood stream when taking a blood meal. Many patients are asymptomatic, but infection can lead to major debilitating conditions, including lymphedema, which causes swelling of arms, legs, breasts and genitalia, and hydrocele, which causes swelling of the scrotum in males [16], [17]. It has been estimated that approximately 13% of infected people suffer from the first condition while up to 21% of males living in endemic areas may experience hydrocele. As a result, and following the conclusion by an independent International Task Force for Disease Eradication that lymphatic filariasis may be one of only six infectious diseases that can be considered to be “eradicable” or “potentially eradicable” [18], the World Health Assembly in 1997 adopted Resolution WHA50.29 calling for the elimination of LF as a public health problem globally.

Although attempts have been made in the past to map the geographic distribution of LF in Africa, this has either been based on simply displaying infected sites as points or as ranges interpolated between such points on local-level maps [19], [20], [21], [22], [23], [24], [25], geostatistical modelling of point prevalence data at regional levels [10], [26] or mapping of aggregated levels of infection at various within and between country or regional levels [9], [10], [27]. The exception has been the work of Lindsay and Thomas [8], who used published community LF prevalence data in conjunction with climate layers and a logistic regression model to predict the distribution and refine the first estimates of the population at risk for LF across sub-Saharan Africa [9].

These statistical modelling approaches have been important in describing and delimiting geographic ranges of species distributions; however, recent studies have highlighted several limitations of applying these models to mapping parasite distributions. First, simple statistical models, such as logistic regression, are restricted because they often fit linear functions between environmental variables and presence/absence data, when it is most likely that such associations are highly complex and non-linear [11], [28]. Second, it is also difficult using these methods to address complex interactions between such variables [29], [30]. Finally, using absence data in logistic regression modelling of LF distribution is complicated by the unreliability of such data owning to the use of variable blood volumes for diagnosing mf infection [31]. The key problem here is that any “absence” record may either represent a true absence of infection (implying non-suitability of location) or arise as a limitation of parasite detectability, whereas if infection is recorded as being present in a location, it is fairly certain that it occurs there.

Here we adopt a machine learning approach that allows flexible modelling of complex non-linear dependencies between infection presence and predictor variables in multidimensional space. This allows us to better understand the ecological niche and to construct a more reliable map of the potential spatial distribution of LF [30], [32], [33], [34]. Such ecological niche models predict the geographic range of a disease or species by: (1) extracting associations between presence data and environmental covariates, (2) using these relationships to characterise the environmental requirements of the species, and (3) deploying this information to predict suitable habitats over unsurveyed areas. This approach has traditionally been used to predict the geographic range of species [34], [35], but more recently it has been used to model the distribution of diseases [36], [37], [38].

There are currently a wide array of algorithms that can be used to model species' ecological niches using machine learning approaches [39], [40]. In this study, we evaluated Maxent, a presence-only maximum entropy-based niche modelling technique [41], to describe the ecological requirement and current potential distribution of LF in Africa, and to determine for the first time how future climate change may affect the distribution and burden of this disease on the continent so that better prevention and control efforts could be directed to mitigate against the effects of such change.

## Methods

### LF Occurrence Data

**Figure 1:**
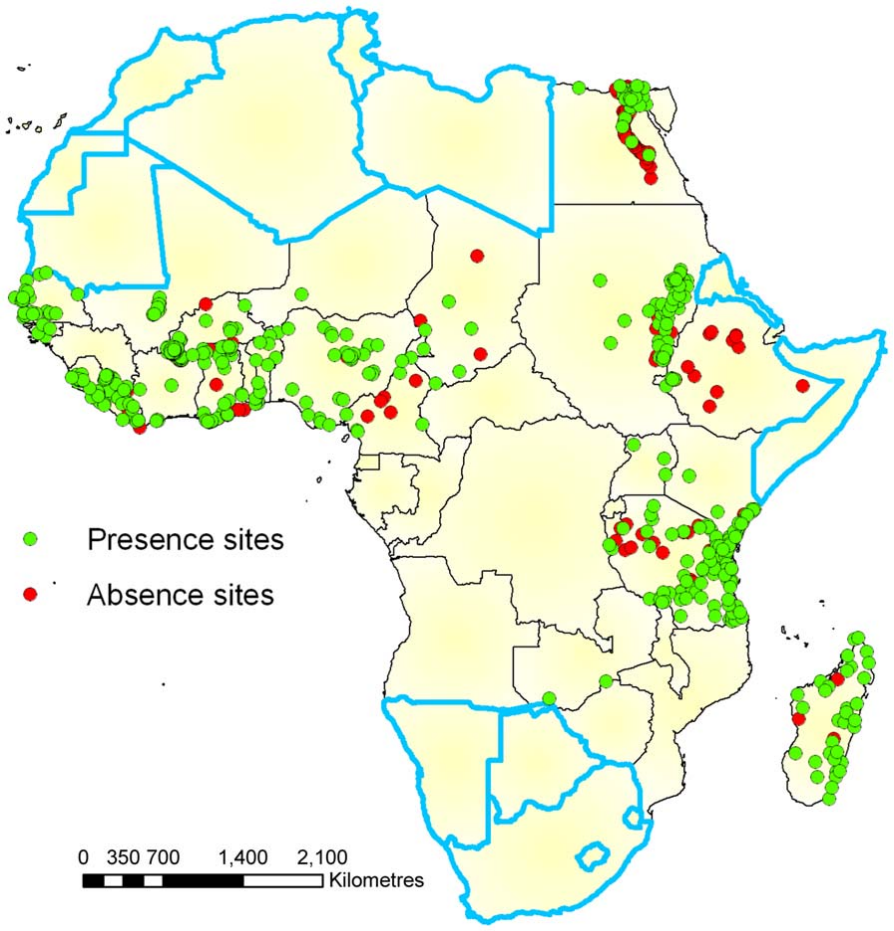
Locations of study sites. Green points show sites where LF infection were found to be present and red sites show sites where it was absent. Non-endemic countries are outlined in blue.

Point data for LF occurrence or presence were collated from community surveys published in the research literature dating from 1940 to 2009, using the online and manual search procedures described in Michael *et al.* [17]. Studies were selected if the surveys described the number of people surveyed, the number positive for microfilaraemia, and were conducted at a specific community site. We found a total of 664 community-specific datapoints of which 508 comprised presence data. These were used in the present analysis (see details of selected studies in Appendix S1 in Supporting Information). Geo-coordinates for each chosen datapoint were either referenced from information given in the literature or by using Google Earth (see Figure 1). We were unable to find latitude and longitude details for 19 of these data points, while geo-coordinates for approximately 21% of the data locations used were only expressed to 2 decimal places.

### Environmental Layers

We initially selected ten environmental and demographic variables, believed to influence the transmission of LF in this analysis [8]. Population density has not normally been employed as a predictor in most previous studies of pathogen distribution modelling; however, we view it as a key determinant of the potential distribution of LF for two reasons: 1) it is a component of the basic reproduction number for vector-borne diseases, such as LF, which determines the extend of spread and prevalence of such diseases, and 2) LF can only occur in inhabited places as the humans are the only host reservoir of the LF parasite in Africa [37], [42], [43], [44].

The use of interpolated climate data or remote sensing data in combination with advanced statistical techniques to map the distribution of vector-borne diseases has accelerated greatly over the last 25 years [45], [46]. Interpolated climate data layers are created by collecting large amounts of weather station data which are then processed to produce continuous climate maps using various smoothing algorithms. One of the most commonly used interpolated global climate data resource is WorldClim (www.worldclim.org) [47]. The WorldClim data are a set of climate data layers of the whole world available at resolutions of around 1 km, 5 km, 9 km or 18 km. The variables available are monthly mean, minimum and maximum temperature and monthly precipitation, and 19 derived bioclimatic variables. The WorldClim layers representing current climate conditions are smooth maps of averaged monthly climate data obtained over the period 1950–2000 from thousands of weather stations (47,554 locations for precipitation data, 24,542 for mean temperature, and 14,835 for minimum and maximum temperature – www.worldclim.org). The data have been interpolated down to a 30 arc-second high resolution grid (often referred to as “1 km^2^” resolution) using a second-order thin plate smoothing spline with altitude, longitude and latitude as independent variables (Hijmans et al. 2005). Uncertainty in the data can arise from inaccurate weather station data or from the interpolation method – this second effect will be magnified in areas with sparse weather station data. For example, while precipitation data are fairly densely distributed in Africa, temperature data is much sparser. There are also very few data points in areas with low population density, particularly in the Sahara and Central Africa (Hijmans et al. 2005). These heterogeneities mean that such data and modelling uncertainties must be taken into consideration when assessing the accuracy of the predictions from the Maxent model.

The worldclim dataset is useful for infection mapping as the data are freely available on a small spatial scale. The data can be used to create new data layers, for example minimum temperature in the coldest month, or maximum temperature in the hottest month, to represent the temperature extremes in a region that could be important for vector and parasite dynamics [48], [49]. One major drawback is that the climate surfaces represent average temperature or precipitation over a period of time, and hence there is no indication of the annual variability which could have a major impact on transmission dynamics.

Altitude data for this study were also obtained from www.worldclim.org – these data were collected by http://www2.jpl.nasa.gov/srtm/ and produced from data collected by a radar system circulating the earth to create a high resolution map of the globe. Similarly, NDVI data were downloaded from http://edit.csic.es/GISdownloads.html; these maps were originally obtained from satellite images (NOAA-AVHRR) over the entire globe. Twelve monthly NDVI maps are available, each of which represents the mean monthly NDVI over an 18 year period from 1982 to 2000. We averaged these maps to produce an annual mean NDVI map. Population density data was created using data from, amongst others, the Socioeconomic Data and Applications Center (SEDAC) at Columbia University (http://sedac.ciesin.columbia.edu/gpw/). These data are created by interpolating global census data to create smooth population maps which are then scaled to match United Nations totals.

The data had slightly different spatial scales (worldclim data ∼9 km^2^, NDVI ∼12 km^2^ and population density ∼5 km^2^), and so were resampled using ArcGIS to give all the layers the same grid size. This resulted in a scale of around 12 km^2^.

### Ecological Niche Modelling

The ecological niche of a species can be defined as those ecological conditions under which it can maintain populations without immigration [50]. Ecological niches and associated potential geographic ranges can be approximated using correlative algorithms that by relating known point-occurrence data to digital GIS data layers, summarize spatial variations in these layers in multidimensional environmental space [51]. Here, we used the maximum entropy method as implemented by the Maxent software to derive the ecological niche for LF occurrence in Africa. We initially compared the performance of Maxent with another widely used modelling package GARP [52]. Maxent was selected for further use in this study as it performed better in tests of model predictive ability (Appendix S2).

Maxent is a general-purpose machine learning programme and has been widely used to predict species distributions [33], [41], [53], [54]. The maxent algorithm essentially builds ecological niche models by quantifying the unknown probability distribution defining the occurrence of a species across a study area without inferring any unfounded information about the observed distribution. The approach aims to find the probability distribution of maximum entropy (that which is closest to uniform) subject to constraints imposed by the observed spatial distributions of the species and environmental conditions. Maxent thus outputs the maximum entropy distribution that satisfies these constraints, thereby providing the least biased description for a given dataset [41], [55]. We implemented Maxent models using version 3.3.1 of the software developed by S. Phillips and colleagues (http://www.cs.princeton.edu/~schapire/maxent/). Selection of the convergence threshold and regularization values was carried out following default rules and the number of iterations was chosen such that all models converged. The default logistic model was used to ensure that predictions gave estimates between 0 and 1 of the probability of infection presence per map pixel.

### Performance Measures

The performance of a model predicting the potential distribution of species presence is traditionally assessed by calculating the area under the curve (AUC) of the receiver operator characteristic (ROC) [56]. This is a plot of the sensitivity (the proportion of correctly predicted known presences, also known as absence of omission error) vs. 1-specificity (the proportion of incorrectly predicted known absences or the commission error) over the whole range of threshold values between 0 and 1. The model AUC thus calculated is compared to the null model which is an entirely random predictive model with AUC = 0.5, and models with an AUC above 0.75 are normally considered useful [57]. Using this method, the commission and omission errors are therefore weighted with equal importance for determining the performance of the model. However, for a presence only ecological niche model this method may be unsuitable for two key reasons [58], [59]: 1) we are less interested in the performance of the model over all the whole of the ROC space, for example, where the omission or commission error is very high, and 2) as we do not have absence data, Maxent simulates pseudo absence data which are drawn at random from the training region. Since these do not represent true absences, mispredicting a known presence may be a more serious failing of the model than mispredicting a possible absence because while the presences are known, the absences are ‘guessed’. In addition omission error has been shown to provide a better metric than commission errors for assessing model fit [60].

**Figure 2:**
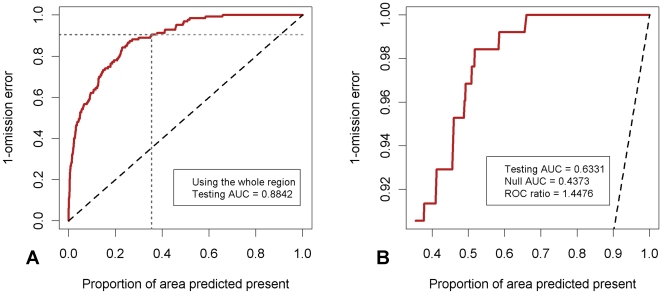
Comparison of traditional versus partial receiver operating characteristic (ROC) curves for the Maxent A model. A) Traditional ROC curve. The horizontal dash line indicates the region of interest for the partial AUC plot – where the omission error is less than E (in this case E = 10). B) The partial AUC plot. The dashed line indicates the null model.

For these reasons, we carried out the analysis of model performance using the partial AUC procedure as described in Peterson *et al.* [59]. The criticisms raised above are answered using this method by: 1) using only presence data (not pseudo absence data) and 2) introducing a user defined variable E which refers to the maximum allowable level of omission error. The ROC curve is now a plot of sensitivity versus the proportion of the study area predicted as present. Only the region where the omission error is less than E is considered. The partial AUC is then a ratio of the AUC of the restricted ROC curve to the AUC of the restricted null model line (see Figure 2 and Peterson *et al.* [59] for full details of this method). The partial AUC was calculated using Simpsons trapezium rule via routines implemented in R. We closely examined two levels of omission error, E = 100 which is essentially a traditional ROC plot as we are assessing the model over all levels of omission error, and E = 10 where we assume that 10% of the positive predictions are actually negative, ie., we are only concerned with assessing models where omission error is less than 10%. Note that overlooking specificity could have significant effects on model accuracy as well as the predicted prevalence of infection (the overall proportion of locations where infection is predicted to be present). This outcome, however, is unlikely to be a major problem for the present study given that 76% of the surveys in our overall dataset (see Methods) reported positive LF infection, with analytical studies showing that at this moderately high level of prevalence specificity issues may have low significance for binary classification [58]–[63].

### Model Implementation

The data were split into two groups: 75% was used to construct the model and form the functional relationships between presence and the environmental variables, and the remaining 25% was used to test the predictive ability of the model. The training region was chosen to be all the countries that are thought to be LF endemic, and the resulting model was projected over the whole of Africa. We assessed model performance by considering the partial AUC values of the testing data. We estimated the error associated with these values by performing a bootstrap algorithm, where we sample with replacement from the testing data 200 times and calculate the partial AUC for each sample.

Maxent has five feature classes (linear (L), quadratic (Q), product (P), threshold (T) and hinge (H)) that can be used to model the functional response of presence probability to changes in the environmental variables [41], [54]. We experimented with using different combinations of features to produce the best performing model. Some of the explanatory layers are also likely to be more predictive than others. We thus aimed to find a set of variables that are predictively powerful and independent as possible. We employed two techniques to determine the most important variables: 1) by considering the percentage contribution that each variable made to the total test gain; and 2) by determining which variables caused the biggest lost in AUC when the data was resampled using a jackknife procedure where one variable was excluded at a time.

In addition, a quadratic discriminant analysis (QDA) was carried out in R to explore how interactions between the identified climate variables determine areas of LF presence or absence. Discriminant analysis essentially seeks to assign data into a series of discrete groups or classes based on the characteristics (**X**) of each data point, such that the probability of correct classification is maximised. QDA extends simple Linear Discriminant Analysis by allowing the intraclass covariance matrices to differ between classes, so that discrimination is based on quadratic rather than linear functions of **X**. In our case, we used QDA to classify presence and absence data correctly based on the climatic conditions of each point.

### Estimating populations at risk

We estimated the number of individuals at risk by overlaying a LF binary risk map on a population map and calculating the population in the ‘positive’ at-risk cells. The SEDAC 2010 population layer for Africa was used for calculating the current at-risk population (http://sedac.ciesin.columbia.edu/gpw/global.jsp). Note that climate data for 2010 was unavailable, and we were therefore forced to use the data averaged between 1950 and 2000 for making these estimations. We constructed the LF binary layer by converting the continuous risk maps produced by Maxent into areas that are suitable and unsuitable by defining thresholds below which the probability of LF occurrence is considered to be zero and above which the probability is considered to be one. Traditionally these classification thresholds are determined by selecting the value that a) maximises the sum of sensitivity and specificity [61], b) where commission error = omission error [62] or c) is equal to the lowest predicted probability at a training presence site [41], [63]. However, methods a) and b), as noted above, assume equal importance of omission and commission errors, and method c) is not suitable when we have an accepted level of omission error. When E = 100 we adopt the lowest training presence threshold approach, and when E = 10 we use a slightly modified version of c) suggested by A.T. Peterson (personal communication), where we take the threshold to be the value of the predicted probability from the E^th^ quantile of the values at training data sites (ie. when E = 10 we use the 10^th^ percentile training presence value).

### Future LF Predictions

The future potential distribution of LF was estimated by using the current Maxent model to make projections over projected climate and population density for 2050. The future climate data were downloaded from www.worldclim.org. These layers were constructed using data from general circulation models (GCMs). The IPCC report [64] considers around 25 GCMs and several emissions scenarios. The temperature projections amongst all the climate models are fairly consistent, however, there is much more uncertainty regarding precipitation. In this study, we consider just two of these GCMs - the Hadley Centre global climate model HADCM3 and the Canadian Centre for Climate Modelling and Analysis model CCCMA under two IPCC climate scenarios – A2a and B2a [65]. A2a is a more extreme scenario, assuming massive disparities between regions in high population growth and energy use, whereas B2a aims to capture a less disparate world with efforts focused towards social equity; this scenario also assumes lower population and economic growth than A2a. To account for differences in population growth between the two climate scenarios we multiplied the 2000 population data by country specific UN medium variant population growth rate predictions for the B2a scenario and by the high variant growth rate predictions for the A2a scenario (http://esa.un.org/unpp/).

Note that WorldClim provides projected future climate data (for years 2020, 2050 and 2080) at four spatial resolutions; 30 seconds (∼1 km^2^ spatial resolution), 2.5 minutes, 5 minutes, and 10 minutes (∼344 km^2^ resolution). These data have been produced with a simple downscaling technique from the coarser resolution predictions of climate models. In this procedure, projected changes in a climate variable, specifically the absolute or relative differences between outputs of a GCM simulation for the baseline years (typically 1960–1990 for future climate studies) and the simulated target years (eg. 2050), are first developed. Then, these changes are interpolated to grid cells with 30 arc-second resolution, with the assumption made that the change in climate is relatively stable over space (ie. has high spatial autocorrelation). Finally, these high resolution changes are applied/calibrated against interpolated observed climate data of the current period (WorldClim data set) to get high resolution projected climate data of the target year.

## Results

### Model Selection

Maxent models can be run with any combination of five feauture classes or real-valued functions, *f_1_*,…*f_n_* on environmental variables, *X* (viz. linear, quadratic, product, threshold and hinge. We initially ran a series of models using different combinations of these feature classes (L,Q,P,T,H) and selected three candidate models with the highest testing partial AUC values to investigate further. Model A employed the quadratic and threshold features, model B used the linear and threshold features, and model C used all the feature classes.

The relative importance and contribution of the original ten environmental, altitude and population density variables to the initially selected three niche models of LF occurrence, assessed by considering the percentage contribution that each variable made to the total test gain and by using a jackknife procedure to determine which of these variables caused the biggest lost in model AUC when each was excluded one at a time, resulted in the selection of the following five variables: population density, mean maximum temperature, mean temperature in the coldest month, mean annual precipitation and altitude. Together they accounted for more than 88% of the total test gain. Specifically, these were selected by firstly excluding the variables which performed poorly using both methods: NDVI, annual mean temperature, mean temperature in the warmest month, and secondly, by identifying the most correlated variables (mean temperature in the coldest month and mean minimum temperature (0.92), and precipitation in the wettest month and mean annual precipitation (0.95)), and selecting the best performing variable from each pair. These were mean temperature in the coldest month as it contributed more than twice as much to the test gain and performed similarly using the jackknife test, and mean annual precipitation as it added slightly more to the test gain and caused a bigger loss in AUC when excluded using the jackknife test.

The three selected models were rerun with the new set of five explanatory layers and model performance was assessed using two different levels of acceptable omission error. This showed that model A, which uses quadratic and threshold features (Table 1), has a slightly higher combined testing partial AUC and the highest entropy. Figure 2 compares the partial AUC plot (E = 10) for model A (Figure 2b) against the whole AUC plot (E = 100) (Figure 2a) with 1- omission error depicted on the y-axis and the proportion of area predicted positive on the x-axis for both plots.

**Table 1:** Summary results from the three Maxent models tested in the present analyses.

Model (Features)	E = 10	E = 100	Entropy	Environmental Variables (% contribution)	Features
A (Q+T)			**8.2938**	Population density 57.3	Q,T
	**1.4656**	**1.7622**		Altitude 26	Q,T
	1.3331, 1.5927	1.6934, 1.8336		Mean temp in coldest month 6.7	Q,T
				Mean annual precipitation 5.2	Q,T
				Mean max temp 4.8	Q,T
B (L+T)			**8.2921**	Population density 57.3	L,T
	**1.4562**	**1.7646**		Altitude 25.9	L,T
	1.3315, 1.6057	1.6762, 1.8322		Mean temp in coldest month 6.8	L,T
				Mean annual precipitation 5.3	L,T
				Mean max temp 4.8	L,T
C (All)			**8.2577**	Population density 57	All
	**1.4227**	**1.7455**		Altitude 25.4	Q,P,T,H
	1.3278, 1.5600	1.6461, 1.8249		Mean temp in coldest month 6.8	L,P,T,H
				Mean annual precipitation 5.9	L,P,T,H
				Mean max temp 4.7	L,P,T,H

See text for explanations of terms. Model A with quadratic and threshold features was selected as it performed the best using the two E – the acceptable level of omission error – thresholds used in this study.

The relative contributions of the explanatory variables to the different Maxent models (assessed using the jackknife procedure) is shown in Table 1. The results indicate that population density contributed the most (up to 57%) to each of the tested models, followed by altitude (around 26%) as the next most significant factor. For model A, the three climatic layers contributed in total to around 17% of the overall prediction of LF occurrence. All our final models performed significantly better than the null model (all partial AUC's >1.42), re-emphasizing the high predictability that can result from ecological niche modelling using the Maxent programme [41], [66].

### Model Predictions

**Figure 3:**
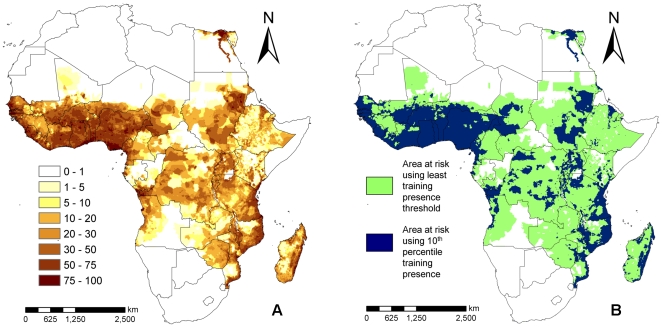
Probability and infection binary maps of the current occurrence of LF in Africa as predicted by the final Maxent A model containing the variables: mean annual precipitation, mean maximum temperature, and mean temperature in coldest month, altitude and population density, and quadratic and threshold features. A) Probability map where probability of occurrence is depicted in the form of percentages. B) LF binary map showing areas with and without infection presence for E = 100 (ie. using classification value from the least training presence threshold) and E = 10.

The distribution of LF occurrence in Africa predicted by the best performing Maxent model (A) is shown in Figure 3. The map shows that LF in Africa occurs over a large area extending from the west to the east primarily across the middle region of the continent. The results also depict a high degree of heterogeneity in the probability of LF occurrence on the continent. There appears to be a large zone exhibiting a high probability of LF occurrence in the Western Africa region, whereas in Central and Eastern Africa and in Madagascar, large areas of medium probability are interspersed with smaller areas of high probability, especially along the coasts. Importantly, all LF-free countries (as shown in Figure 1) are shown to have fairly low probabilities of infection. Most of the training data are located in west and east Africa and there are very few datapoints covering central Africa. Little is known about the state of LF in many of these countries, meaning we have no way to validate the model in these regions. For this reason, we need to be cautious when interpreting the results from these countries compared to more densely sampled countries.

**Figure 4:**
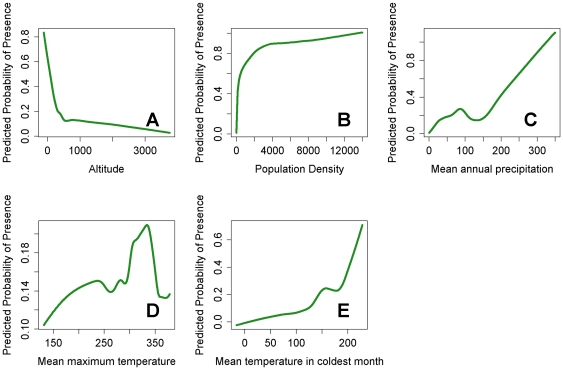
Graphs showing the marginal relationships between each environmental variable and the probability of LF occurrence. Temperature values are expressed in ×10°C, precipitation is in *mm* per month and altitude is in metres.

Individual response curves (marginal responses obtained by keeping all other variables at their average sample value) of the relationships between each environmental variable and the probability of disease occurrence as estimated by model A are portrayed in Figure 4. The results clearly exhibit complex but quadratic relationships between each of the best five environmental/population drivers and probability of LF occurrence. In general, however, there is an overall negative response observed between altitude and LF occurrence and nonlinear positive responses observed for the rest of the variables. There also appears to be evidence for threshold effects in each of the estimated relationships (most clearly observed for the association between mean temperature in coldest month and probability of LF infection (Figure 4e)), wherein the probability of LF occurrence begins to increase only after about 10°C).

**Figure 5:**
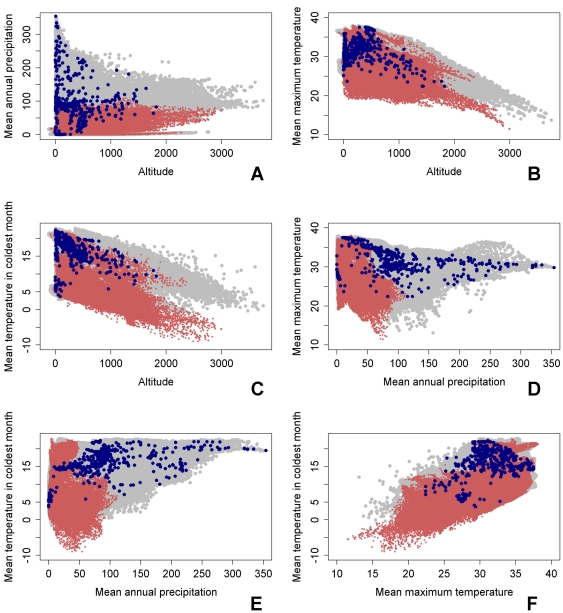
Visualizations of the modelled LF ecological niche in Africa. The grey points are the environmental conditions for every cell in Africa, the red points represent the conditions in non-endemic sites while the conditions underlying presence sites are shown in blue. Temperature values are expressed in ×10°C, precipitation is in *mm* per month and altitude is in metres.

To visualise the LF ecological niche in Africa, the Maxent predictions were further related to environmental conditions at both presence sites and areas where the disease is known not to exist (Figure 5). The two-dimensional plots in the figure show that differences in the identified ecological conditions may strongly influence the probability of LF infection presence and absence. These results indicate that LF occurs mainly in the hot and wet regions of Africa, with non-endemic areas all having an annual rainfall level below around 100 mm. The mean maximum temperature and mean temperature in the coldest month both need to be relatively high for the disease to occur, with no presence sites occurring when the temperature in the coldest month is 3.7 degrees and the mean maximum temperature in 22.4 degrees.

**Figure 6:**
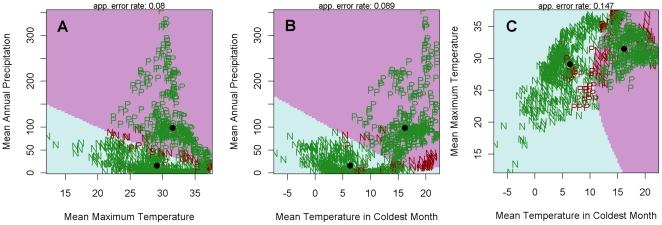
Results from the quadratic discriminant analysis used in this study to determine the importance of the climatic variables included in the final Maxent A model in classifying positive and negative LF occurrence sites in Africa. The purple area of the graph shows the area where infection is present, while the blue portion shows where it is absent. The green points represent correctly classified datapoints and the dark red points show incorrectly classified data points. 91% of the points were classified correctly.

Results from the quadratic discriminant analysis of the contribution of key environmental variables to LF occurrence are shown in Figure 6. These highlight not only that different regions of each variable space can determine where LF is likely to occur and not occur, but also the dependency of such classification on variable interaction. Thus, the levels of rainfall and temperature required for the disease to occur are dependent on each other, whereby in warmer regions, less rainfall is needed to sustain parasite transmission. However, a key finding is that the minimum threshold for mean temperature in the coldest month is around 11 degrees with apparently little variation in this value with increasing mean maximum temperature (Figure 6c).

### Future Climate Predictions

**Figure 7:**
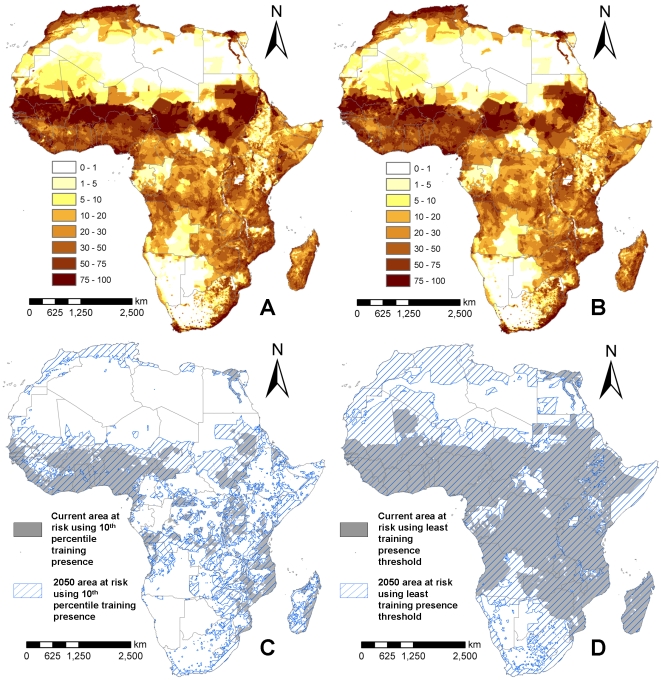
2050 predictions of the distribution of LF using HADCM3 A2a and medium population increase scenario (A), and HADCM3 B2a and high population increase scenario (B). Model predictions for the CCCMA climate predictions are very similar to those shown here. (C) shows the predicted at-risk areas in 2000 (grey) and in 2050 (stripped) for the a2a scenario using a threshold value of 29.8% given by setting the acceptable omission error to E = 10. (D) shows the 2000 (grey) and 2050 (stripped) areas at risk from LF obtained with the a2a climate scenario and using the LTP threshold, with an associated threshold value of 1.9% (see text).

We used model A in conjunction with the four climate change projections and their associated population growth estimates outlined earlier to investigate how the potential distribution of LF could change between now and 2050, assuming that no control measures are implemented. Our model predictions shown in Figure 7 indicate that LF occurrence could increase in large parts of Africa with the highest increases expected in areas bordering the current northern extent of the disease, particularly across regions of Mauritania, Sudan, and Somalia. LF occurrence is also predicted to increase in countries in the southern parts of the continent. The probability of disease occurrence could, however, decrease in other areas, mainly in the west near Ivory Coast and Nigeria and also the Democratic Republic of Congo (Figure 7). Overall, the mean change in probability of LF occurrence over the whole continent was found to be 0.1, suggesting that LF transmission is likely to increase in Africa as climate changes.

### Estimating current and future populations at risk to LF

The populations at risk were estimated in this study by converting the Maxent prediction from model A into a binary map using two thresholds – the value of the least training presence (LTP) prediction which was 1.9% and the value of the 10^th^ percentile of the training presence (10% TP) predictions, which was 29.8%. For each threshold, each cell in the map with a value above these values was deemed to as having LF present. The threshold map for 2010 is shown in Figure 3b and for 2050 in Figures 7c and 7d. The current (2010) population at risk to LF in Africa is calculated to be 804 million using LTP threshold (E = 100) and 542 million using the 10% TP (E = 10) threshold. The 2050 estimates range from 1.86 billion to 1.46 billion using the LTP threshold and from 1.65 billion to 1.30 billion using the 10% TP threshold (Table 2). On average, the A2a scenarios predict a larger at-risk population, indicating that 13% more people would potentially live in at-risk areas when compared with the effects of the B2a scenario.

**Table 2:** Population at risk from LF in endemic countries in Africa estimated applying two different threshold values based on different levels of acceptable omission error, E, to the final Maxent model A predictions.

	Population increase		Least training presence threshold			10th percentile training presence threshold		
	2010–2050							
Country name	medium	high	2010	2050 a2a	2050 b2a	2010	2050 a2a	2050 b2a
Angola	2.23	2.51	15.77 m	42.34 m	37.29 m	4.27 m	18.86 m	17.56 m
Benin	2.39	2.69	7.42 m	20.86 m	18.54 m	7.38 m	20.8 m	18.53 m
Burkina Faso	2.51	2.81	15.71 m	45.07 m	40.22 m	15.26 m	45.06 m	40.22 m
Burundi	1.74	1.97	8.76 m	16.76 m	14.8 m	5.35 m	9.16 m	9.08 m
Cameroon	1.84	2.1	18.13 m	38.48 m	33.79 m	13.3 m	31.29 m	27.61 m
Central African Rep.	1.69	1.95	4.04 m	8.19 m	7.1 m	0.82 m	4.13 m	3.41 m
Chad	2.41	2.7	10.51 m	28.91 m	25.78 m	7.01 m	28.28 m	24.93 m
Congo	1.83	2.08	3.96 m	7.77 m	6.94 m	3.02 m	6.14 m	5.5 m
Djibouti	1.67	1.89	0.57 m	1.2 m	1.07 m	0.42 m	1.01 m	0.89 m
Egypt	1.53	1.77	74.71 m	136.27 m	118.09 m	74.11 m	135.39 m	117.34 m
Equatorial Guinea	2.09	2.36	0.53 m	1.23 m	1.09 m	0.37 m	1.23 m	1.09 m
Ethiopia	2.05	2.31	78.08 m	173.5 m	156.7 m	6 m	29.9 m	25.7 m
Gabon	1.65	1.9	1.19 m	2.57 m	2.23 m	0.68 m	1.37 m	1.21 m
Gambia, The	2.15	2.44	1.36 m	3.24 m	2.88 m	1.36 m	3.24 m	2.88 m
Ghana	1.86	2.1	23.67 m	47.64 m	43.27 m	23.67 m	47.33 m	43.07 m
Guinea	2.32	2.62	9.83 m	25.57 m	22.68 m	4.96 m	22.61 m	18.36 m
Guinea-Bissau	2.16	2.42	1.43 m	2.91 m	2.65 m	1.41 m	2.88 m	2.63 m
Ivory Coast	2.01	2.29	18.3 m	41.48 m	36.99 m	17.39 m	40.37 m	36.2 m
Kenya	2.09	2.39	36.23 m	93.34 m	81.86 m	12.21 m	37.78 m	36 m
Liberia	2.16	2.45	4.52 m	9.35 m	8.41 m	4.33 m	9.28 m	8.34 m
Madagascar	2.12	2.42	20.44 m	48.95 m	42.91 m	13.47 m	41.57 m	38.25 m
Malawi	2.33	2.64	13.95 m	39.34 m	34.7 m	12.68 m	38.96 m	34.25 m
Mali	2.12	2.39	15.02 m	34.32 m	30.5 m	11.52 m	33.45 m	29.29 m
Mozambique	1.89	2.16	20.7 m	47.2 m	41.65 m	16.71 m	45.08 m	39.86 m
Niger	3.66	4.04	14.91 m	62.34 m	56.3 m	10.57 m	60.1 m	52.75 m
Nigeria	1.83	2.06	143.97 m	288.68 m	257.45 m	139.88 m	286.8 m	255.36 m
Rwanda	2.15	2.42	9.44 m	23.8 m	21.16 m	3.7 m	11.74 m	11.67 m
Senegal	2.03	2.3	9.75 m	20.71 m	19.03 m	9.16 m	20.71 m	19.02 m
Sierra Leone	2.13	2.42	5.9 m	13.12 m	11.67 m	5.71 m	13.03 m	11.65 m
Sudan	1.76	2	37.47 m	75.98 m	66.58 m	20.24 m	65.67 m	53.55 m
Tanzania	2.43	2.75	41.79 m	120.35 m	106.21 m	24.05 m	98.13 m	88.77 m
Togo	1.95	2.22	5.57 m	12.39 m	10.93 m	5.57 m	12.36 m	10.93 m
Uganda	2.7	3.04	32.52 m	97.25 m	86.75 m	25.32 m	83.56 m	76.17 m
Dem. Rep. of Congo	2.17	2.45	70.73 m	165.66 m	147.09 m	33.99 m	113.17 m	97.77 m
Zambia	2.18	2.48	12.67 m	32.73 m	28.84 m	2.25 m	21.99 m	18.53 m
Zimbabwe	1.75	2.04	14.9 m	26.04 m	22.44 m	4.76 m	21.38 m	19.16 m
Total			804.42 m	1,855.52 m	1,646.58 m	542.88 m	1,463.8 m	1,297.54 m

2010 populations at risk are calculated by projecting the derived Maxent model against 2000 climate data (but using 2010 population data (see text)) while the 2050 populations at risk are derived using the predicted 2050 environmental/population density data. All figures are expressed in 000’s. Also given are the UN predicted increases in population, shown in terms of the factor s by which national populations are expected to increase.

## Discussion

We have used an ecological niche modelling approach based on infection presence-only data to firstly reveal the spatial distribution of LF in Africa, and the environmental determinants that underlie this pattern, and secondly to investigate how climate change may affect the future potential distribution and burden of this important parasitic disease on that continent. The performance of the Maxent models developed here were assessed using the partial AUC measure, a modification of the usual AUC tool used for evaluating the accuracy of ecological niche models. The benefits of this method over a traditional AUC approach are that it: 1) eliminates the used of pseudo absence data in accuracy measurements, and 2) allows the user to define an acceptable level of omission error.

The advantages of using machine learning approaches, such as the maximum entropy modelling algorithm implemented in the Maxent programme, over simpler statistical tools, such as logistic regression, for species distribution modelling have been thoroughly reviewed previously [30], [39], [41]. Here, we highlight that two chief benefits of applying such methods to parasitic infection mapping arise from their flexibility in specifically accounting for: (1) the complex non-linear associations of infection occurrence with individual explanatory variables, and (2) the impact that interactions occurring among these variables may have on infection presence. This flexibility has provided new insights as to how climate variables may functionally influence LF presence in Africa.

Thus, for example, although the relationship between the probability of LF presence and mean annual precipitation was the least non-linear (Figure 4c), its impact on infection probability is found to be low below a threshold of around 150 mm per year. Biologically, this may be because a certain amount of water is needed to provide suitable laying sites for LF vectors. However, it has been suggested that vector survival can also be affected if there is too much rainfall as egg laying sites can get washed away [67]. If this is true, then our result might imply that such washouts will occur only at precipitation levels above 350 mm. Similarly, the LF occurrence - mean maximum temperature response curve (Figure 4d), is found to increase until it peaks between 25°C and 32.5°C, after which it begins to decrease suggesting this temperature range is the most suitable for LF transmission. This result is consistent with experimental findings that both mosquito survival [68] and the development of LF larvae within the mosquito [49] peak around 22–34°C. Although different measures of temperature were used, it is also consistent with the previous findings of Lindsay and Thomas [8], who found that the temperatures of sites in Africa with microfilaraemic individuals lie within the range between 22 to 30°C. However, our results also indicate that mean temperature in the coldest month (Fig. 4e) could induce the most non-linear effect on LF presence, showing that at temperatures <5°C, the probability of disease presence is almost zero but above this threshold to at least 22°C, a dramatic positive impact on parasite occurrence may occur. These findings suggest that fluctuations in temperature limits rather than mean temperature may represent the key temperature-related bioclimatic thresholds important for supporting LF transmission. In contrast to the effect of climate variables, the relationship between altitude and LF occurrence was found to be negative, although again the association was distinctly non-linear (Figure 4a). Such negative correlations between infection presence and altitude have been recorded previously in field studies [70], [71], and most likely reflect the negative effect of falling temperature with increasing altitude (ie. the lapse rate) on mosquito survival rate and the rate at which the parasite develops within the vector [49], [70].

Exploration of the Maxent modelling results has also allowed a first depiction of how subtle interactions between key climatic variables may govern the suitability of a geographic region for LF transmission to occur. The key finding here is that levels of precipitation and temperature in particular could interact strongly to define the multivariate space required for the disease to occur, with generally less rainfall needed in warmer regions to sustain parasite transmission and vice versa (Figure 5 and 6). The biological significance of this finding is that such interactive effects could result in compensatory responses among vector and parasite ecological traits (vector birth, survival and biting rates, and larval development rate in the vector) that would not only dampen the effects of variations in individual key climatic variables but also allow the transmission of LF to occur over a much wider area than would be the case if habitat suitability is defined solely by each single variable. However, the results also show that an important absolute limiting factor is that the minimum temperature threshold for mean temperature in the coldest month needs to be around 5°C for transmission to occur.

A major finding of this study is that human population density was by far the most significant variable that may influence LF occurrence in Africa. This supports not only theoretical expectations that host population density (and the attendant mosquito density) is a key driver of the transmission of vector-borne infections [42], but is also biologically intuitive given that the adult parasites live in the host and mosquito vectors have a preference for human blood meals to oviposit and reproduce. This result would suggest that climate variables *per se* may play a smaller role in determining the ecological niche and hence the potential distribution of LF. However, given that the best-fitting Maxent model predicts low probabilities of infection occurrence in the known non-endemic and high altitude regions of the continent despite the presence of significant human populations in these areas (most indubitably via effects on both vector and parasites), it is clear that both population density and environmental variables will need to be included together in any study attempting to model the potential geographic distribution of this or any other parasitic infection [37], [43], [44].

The Maxent model for LF occurrence across Africa generated a risk map giving a probability of infection presence in each location between 0 and 1, with a probability close to 1 indicating sites with the highest risk and possibly levels of infection. Thus, the map shown in Figure 2 provides not only information regarding the potential spatial extend of LF endemicity across Africa but also crudely data on variations in the intensity of transmission that can be expected in different parts of the continent. Based on the variation in relative risk shown in the figure, the highest LF transmission therefore appears to occur in the Western Africa region, whereas infection levels in large parts of Central and Eastern Africa and in Madagascar are predicted to be considerably more heterogeneous, with moderate levels interspersed with smaller areas of high infection occurring along the coasts. Despite the fact that the present Maxent results are based only on presence data, this conclusion is surprisingly well supported by the actual national LF prevalence values estimated for the endemic countries of Africa given in Michael *et al.* [17] and Michael and Bundy [9]. This represents an important technical insight as it suggests that modelling of presence-only data may provide a good approximation to actual levels of parasite transmission intensity in an area [8], possibly due to climate-derived variations in the abundance of the relevant transmitting vector species. This is supported by the remarkable similarity of the African ecological niche maps for *Anopheles funestus* and *A. gambiae*, the two principal anopheline vectors of LF in Africa, developed by Moffett and colleagues [37], [72], with the LF risk map shown in Figure 2.

Estimations of the at-risk population for LF in Africa have varied significantly between previous studies, with recently reported figures appearing to increase over time possibly due to the effects of both increasing population and surveillance. Thus, in 1992, it was estimated that some 113 million individuals lived in endemic areas [73], which increased to 212 million [74] and 396 million by 2009 [75]. These estimates, which are normally made by identifying infection positive districts and calculating the number of people in each of these districts, not only take little account of the spatial variation that exists at the local level [22], [26], [76] but are also highly dependent upon the existence of field surveys covering all relevant endemic areas. By contrast, machine learning-based ecological niche models using presence-only data approximate the realised distribution of a disease [54], and via the derivation of a continuous potential distribution map may offer a more accurate method to determine the true extent of infection and hence actual populations at risk. The additional prospect of being able to use different cut-off disease presence thresholds with this method means that we can also explore the implications of error in the data for quantifying risk and disease burdens. Thus, using a low presence threshold equal to the least training presence, we estimate that 804 m people in Africa may be living in at risk areas, whereas assuming a more stringent 10% omission error, we estimate that some 542 m people may be at risk. Given that the average prevalence of LF (infection and disease) in African endemic countries has been estimated to be around 11% [8], [17], our estimate of the populations at risk also thus suggests that we can expect between 60 and 89 million cases of LF in 2010 on that continent compared to the 51 million and 47 million estimated by Michael and Bundy [9] and Lindsay and Thomas [8] respectively for the years 1990 and 2000.

The future potential distribution and burden of LF in Africa as a result of predicted changes in climate and population growth were produced by using the best-fit Maxent model derived for 2000 and projecting the functional relationships therein onto the two 2050 climate scenarios, i.e. we consider that niche dynamics are static and that climate change will not affect either the form of the biotic relationships governing the vector and the parasite population dynamics or any adaptation by these populations to the new environment [32], [77], [78]. We examined the impact of two 2050 climate scenarios from two different global climate models. The more extreme scenario, A2a, predicted on average 13% more people living in at-risk areas than the B2a scenario. Predictions of the 2050 population at risk range from 1.30 to 1.86 billion (Table 2); although a large component of this increase is a result of population growth, changes in climate are also shown to increase the area of Africa that is suitable for LF transmission. In particular, large regions below the Sahara desert and in Zambia, Zimbabwe and Angola are predicted to have increased probability of LF (Figure 7c,d), suggesting that the ecological niche of infection could increase and extend both northwards and southwards. When interpreting these results attention must be paid to the uncertainty and error associated with the future climate data – both from the GCMs and the downscaling procedures adopted, and the fact that we are only considering two GCMs and two emissions scenarios. These results obviously do not take into account increases in disease control activities on the continent of Africa, which has accelerated greatly since the Global Alliance to Eliminate Lymphatic Filariasis was created in 2000. It also does not take account of the increase in vector control on that continent, primarily targeted at malaria, which will have an impact on LF infection via reductions in vector biting rates and lifespans [79]. Indeed, our predictions of the likely future increase in LF burden argues strongly for strengthening and expanding these interventions even further as an important mitigation strategy to counter the predicted spread and intensifying of this debilitating disease in Africa as population density increases and climate changes.

Although our study has yielded several important and novel insights into the determinants and structuring of the ecological niche and the present and future spatial occurrence of LF in Africa, there are several limitations that need to be borne in mind when interpreting the present results. First, even though ecological niche modelling approaches based on occurrence data alone, such as the Maxent algorithm used in this study, are optimized for predicting the realised or actual (rather than the fundamental) distribution of a species [41], [54], predictions of presence will still be dependent on the sample locations of the available data with any deficiency in sample coverage of all suitable areas able to bias the results. Second, the crude scale of the environmental layers used to construct the Maxent model means that the validity of predictions on small focal spatial scales is questionable. Third, we have no error estimates associated with our predictions – in reality we would expect a heterogeneous error map of model predictions in Africa caused by different levels of error associated with the climate data and model fit, and from the biased distribution of presence data. However, it is hoped that our predictions are fairly robust on the district or country wide scales that are typically used in policy decisions regarding disease control and eradication strategies, especially in countries with more accurate climate data and more LF survey data. We have also used 50-year averaged climate layers to approximate a phenomenon that might have changed in the past decade or so to characterise “current” climate in our analyses.

The above caveats indicate that our application is likely to be at the lower limit of the usefulness of the available data. Although it might be possible to use remote-sensed data to overcome a part of this limitation [45], forward projection of such data to future climates is clearly not possible. Combining correlative spatial modelling approaches with mechanistic models linking climate/environmental and population variables to parasite transmission processes in conjunction with regional climate models, may, on the other hand, provide a more useful solution to improving the detail of spatial predictions [69]. The practical modelling frameworks and tools required for successfully achieving this synthesis is, however, still largely indeterminate. We suggest that resolving these conceptual and methodological issues represents the next major challenge in species, including parasite, distribution modelling.

## Supporting Information

Appendix S1 Details of published data used in the Maxent analysis. The number of data points from each study or review is given in brackets. The list of study references for the data used are given below the table.
(DOCX)

Appendix S2 Comparison of GARP and Maxent model fits. Partial AUC ratios taken across 200 bootstrap replications are shown for omission errors, E, of 1 and 5.
(DOCX)
