# Supplementary material for: Predicting the Current and Future Potential Distributions of Lymphatic Filariasis in Africa Using Maximum Entropy Ecological Niche Modelling
Source: PLoS One. 2012 Feb 16;7(2):e32202. doi: 10.1371/journal.pone.0032202 (PMC3281123; doi:10.1371/journal.pone.0032202)
Supplement: Appendix S1 — Details of published data used in the Maxent analysis. The number of data points from each study or review is given in brackets. The list of study references for the data used are given below the table. (DOCX) [file pone.0032202.s001.docx]

**Appendix S1:**

Details of published data used in the Maxent analysis. The number of data points from each study or review is given in brackets. The list of study references for the data used are given below the table.

| Country | References |
| --- | --- |
| Benin | (Hawking, 1977) (7), (Myung *et al.*, 1998) (3) |
| Burkina Faso | (Brengues, 1975) (3), (Gidel *et al.*, 1969) (2), (Guigemde *et al.*, 1983) (2), (Gyapong *et al.*, 2002) (6), (Hawking, 1977) (1), (Lamontellerie, 1972) (73) |
| Cameroon | (Languillon, 1957) (10), (Moyou-Somo *et al.*, 2003) (1) |
| Chad | (Hawking, 1977) (6) |
| D.R.Congo | (Fain *et al.*, 1974)(1) |
| Egypt | (El-Setouhy *et al.*, 2007) (1), (Mcconnell & Dennis, 1976) (17), (Sasa, 1976) (2), (Shawarby *et al.*, 1965)(90) (Weil *et al.*, 1999) (3) |
| Ethiopia | (Hawking, 1977) (1), (Mcconnell & Dennis, 1976) (1) |
| Gambia | (Knight, 1980) (11), (Sasa, 1976) (2) |
| Ghana | (Dunyo *et al.*, 1996) (6), (Gbakima *et al.*, 2005) (3), (Gyapong *et al.*, 1996a) (10), (Gyapong *et al.*, 1996b) (6) (Gyapong *et al.*, 1998) (4), (Gyapong *et al.*, 2002) (11) |
| Guinnea-Bissau | (Sasa, 1976) (4) |
| Ivory Coast | (Brengues, 1975) (3), (Hawking, 1977) (2) |
| Kenya | (Aburu, 1974) (5), (Mukoko *et al.*, 2004) (9), (Njenga *et al.*, 2000) (4),  (Wamae *et al.*, 2001) (20) |
| Liberia | (Brinkmann, 1976) (3), (Burch & Greenville, 1955)(3), (Chlebowsky & Zielke, 1980) (3), (Hawking, 1957) (1) (Hawking, 1977) (1), (Kuhlow & Zielke, 1976) (46), (Poindexter, 1950) (3), (Sasa, 1976) (1) |
| Madagascar | (Brunhes *et al.*, 1972) (2), (Brygoo, 1958) (34), (Coulanges, 1982) (2),  (Rakotomalala *et al.*, 1995) (1) |
| Malawi | (Ngwira *et al.*, 2002) (6), (Ngwira *et al.*, 2007) (47) |
| Mali | (Brengues, 1975) (11) |
| Niger | (Hawking, 1977) (1), (Sasa, 1976) (1) |
| Nigeria | (Anosike & Onwuliri, 1994) (3), (Anosike *et al.*, 2005) (1), (Engelbrecht *et al.*, 2003) (1), (Onwuliri & Anosike, 1989)(5) (Sasa, 1976) (1), (Terranella *et al.*, 2006) (1), (Udonsi, 1986) (4), (Ufomadu *et al.*, 1990) (12), (Wijeyaratne *et al.*, 1982) (2) |
| Senegal | (Diallo *et al.*, 1977) (2), (Diallo *et al.*, 1983) (1), (Hawking, 1977) (6),  (Juminer *et al.*, 1971)(1) |
| Sierra Leone | (Gbakima *et al.*, 1996) (3), (Gbakima & Sahr, 1996) (5), (Hawking, 1957) (1) |
| Sudan | (Satti & Abdel Nur, 1974) (3) |
| Tanzania | (Hawking, 1940) (1), (Hawking, 1977) (13), (Jordan, 1954) (15), (Jordan, 1956c, a, b) (56), (Sasa, 1976) (4), (Wegesa *et al.*, 1979) (2), (Southgate, 1992) (1), (Simonsen *et al.*, 1995) (2), (Meyrowitsch *et al.*, 1996) (2), (Massaga *et al.*, 2000) (2), (Meyrowitsch *et al.*, 2004) (1), (Jaoko *et al.*, 2007) (1) |
| Togo | (Hawking, 1977) (1), (Scheiber & Braun-Munzinger, 1976) (3) |
| Uganda | (Hawking, 1940) (1), (Onapa *et al.*, 2001)(2) |
| Zambia | (Roberts *et al.*, 1973) (1) |
| Zimbabwe | (Hawking, 1977) (2) |

References

Aburu, D.E. (1974) Studies on filarial infections at the East African coast. Annual report of East African Institute of Malaria and vector-borne diseases, Jan 74 - Dec 75.

Anosike, J.C. & Onwuliri, C.O. (1994) Studies on filariasis in Bauchi state, Nigeria. II. The prevalence of human filariasis in Darazo local government area. *Parasitology*, **35**, 242-250.

Anosike, J.C., Nwoke, B.E., Ajayi, E.G., Onwuliri, C.O., Okoro, O.U., Oku, E.E., Asor, J.E., Amajuoyi, O.U., Ikpeama, C.A., Ogbusu, F.I. & Meribe, C.O. (2005) Lymphatic filariasis among the Ezza people of Ebonyi state, Eastern Nigeria. *Annals of Agricultural and Environmental Medicine,* **12**, 181-186.

Brengues, J. (1975) La filariose de Bancroft en Afrique de l'Ouest. *Memoires d’ORSTOM*, **79**, 1-299.

Brinkmann, U.K. (1977) Epidemiological investigations of Bancroftian filariasis in the coastal zone Liberia. *Tropenmedizin Und Parasitologie*, **28**, 71-76

Brunhes, J., E. , Rajaonarivelo, E. & Nelson, G.S. (1972) Epidemiologie de la filaroise de Bancroft a Madagascar. *Cahiers ORSTOM Serie Entomologie Medicale et Parasitologie*, **10**, 193-205.

Brygoo, E.R. (1958) La filariose humain a Madagascar. *Archives de l'Institute Pasteur de Madagascar*, **26**, 23-39.

Burch, T.A. & Greenville, H.J. (1955) Filariasis in Liberia. *American Journal of Tropical Medicine and Hygiene*, **4**, 47-51.

Chlebowsky, H.O. & Zielke, E. (1980) Studies on Bancroftian filariasis in Liberia, West Africa. III. Efficacy of repeated treatment with diethylcarbamazine and vector control on the microfilarial reservoir in a rural population. *Tropenmedizin Und Parasitologie*, **31**, 181-193.

Coulanges, P. (1982) Data on lymphatic filariasis caused by *w. bancrofti* in Madagascar. *Archives de l'Institut Pasteur de Madagascar (Tananarive)*, **50**, 23-33.

Diallo, S., Sarr, M., Diagne, S. & Konate, L. (1977) Filariasis in the Nianing region, Senegal. Prevalence, clinical aspects, entomological findings. *Medecine Afrique Noire*, **24**, 233-242.

Diallo, S., Bah, I.B., Victorius, A., N'dir, O. & Diouf, F. (1983) Effects of diethylcarbamazine on the transmission of *Wucheria bancrofti* in a Senegalese reservoir of lymphatic filariasis. *Dakar Medical*, **28**, 179-186.

Dunyo, S.K., Appawu, M., Nkrumah, F.K., Baffoe-Wilmot, A., Pedersen, E.M. & Simonsen, P.E. (1996) Lymphatic filariasis on the coast of Ghana. *Transactions of the Royal Society of Tropical Medicine and Hygiene*, **90**, 634-638.

El-Setouhy, M., Abd Elaziz, K.M., Helmy, H., Farid, H.A., Kamal, H.A., Ramzy, R.M., Shannon, W.D. & Weil, G.J. (2007) The effect of compliance on the impact of mass drug administration for elimination of lymphatic filariasis in Egypt. *American Journal of Tropical Medicine and Hygiene*, **77**, 1069-1073.

Engelbrecht, F., Oettl, T., Herter, U., Link, C., Philipp, D., Edeghere, H., Kaliraj, P. & Enwezor, F. (2003) Analysis of *Wuchereria bancrofti* infections in a village community in northern Nigeria: Increased prevalence in individuals infected with *Onchocerca volvulus*. *Parasitology International*, **52**, 13-20.

Fain, A., Elsen, P., Wery, M. & Maertens, K. (1974) Human filariases in the Mayumble region and adjacent areas (Zaire republic). Evaluation of the microfilarial density. *Annales de la Societe Belge de Medecine Tropicale*, **54**, 5-34.

Gbakima, A.A. & Sahr, F. (1996) Filariasis in the Kaiyamba chiefdom, Moyamba district Sierra Leone: an epidemiological and clinical study. *Public Health*, **110**, 169-74.

Gbakima, A.A., Pessima, J. & Sahr, F. (1996) Parasitological and clinical studies on *Wuchereria bancrofti* infection in Moyamba district, Sierra Leone. *African Journal of Health Sciences*, **3**, 37-40.

Gbakima, A.A., Appawu, M.A., Dadzie, S., Karikari, C., Sackey, S.O., Baffoe-Wilmot, A., Gyapong, J. & Scott, A.L. (2005) Lymphatic filariasis in Ghana: Establishing the potential for an urban cycle of transmission. *Tropical Medicine & International Health*, **10**, 387-392.

Gidel, R., Brengues, J. & Rodhain, F. (1969) Evaluation of 2 immunological tests (skin test and complement fixation test) for the detection of filariasis in populations of Upper Volta where *Wuchereria bancrofti*, *Onchocerca volvulus* and *Dipetalonema perstans* occur together. *Bulletin of the World Health Organization*, **40**, 831-842.

Guigemde, T.R., Sokal, C.D. & Roux, J. (1983) Study of filarial associations in two villages in south-western Upper Volta. Medecine Afrique Noire **30**, 411-417

Gyapong, J.O., Adjei, S. & Sackey, S.O. (1996a) Descriptive epidemiology of lymphatic filariasis in Ghana. *Transactions of the Royal Society of Tropical Medicine and Hygiene*, **90**, 26-30.

Gyapong, J.O., Omane-Badu, K. & Webber, R.H. (1998) Evaluation of the filter paper blood collection method for detecting og4c3 circulating antigen in bancroftian filariasis. *Transactions of the Royal Society of Tropical Medicine and Hygiene*, 92, 407-410.

Gyapong, J.O., Adjei, S., Gyapong, M. & Asamoah, G. (1996b) Rapid community diagnosis of lymphatic filariasis. *Acta Tropica*, **61**, 65-74.

Gyapong, J.O., Kyelem, D., Kleinschmidt, I., Agbo, K., Ahouandogbo, F., Gaba, J., Owusu-Banahene, G., Sanou, S., Sodahlon, Y.K., Biswas, G., Kale, O.O., Molyneux, D.H., Roungou, J.B., Thomson, M.C. & Remme, J. (2002) The use of spatial analysis in mapping the distribution of Bancroftian filariasis in four west African countries. *Annals of Tropical Medicine and Parasitology*, **96**, 695-705.

Hawking, F. (1940) Distribution of filariasis in Tanganyika territory, East Africa. *Annals of Tropical Medicine and Parasitology*, **34**, 107-119.

Hawking, F. (1957) The distribution of Bancroftian filariasis in Africa. *Bulletin of the World Health Organization*, **16**, 581-592.

Hawking, F. (1977) The distribution of human filariasis throughout the world. Part iii. Africa. *Tropical Diseases Bulletin*, **74**, 649-679.

Jaoko, W.G., Michael, E., Meyrowitsch, D.W., Estambale, B.B., Malecela, M.N. & Simonsen, P.E. (2007) Immunoepidemiology of *Wuchereria bancrofti* infection: Parasite transmission intensity, filaria-specific antibodies, and host immunity in two east African communities. *Infection and Immunity*, *75*, 5651-5662.

Jordan, P. (1954) Filariasis in the southern province of Tanganyika. East African *Medical Journal*, **31**, 537-542.

Jordan, P. (1956) Filariasis in the eastern, Tanga and northern provinces of Tanganyika. *East African Medical Journal*, **33**, 225-233.

Jordan, P. (1956) Filariasis in the lake province of Tanganyika. *East African Medical Journal*, **33**, 237-242.

Jordan, P. (1956) Filariasis in the western province of Tanganyika. *East African Medical Journal,* **33**, 233-236.

Jordan, P. (1960) Bancroftian filariasis in Tanganyika: Observations on elephantiasis, microfilarial density, genital filariasis and microfilaraemia rates. *Annals of Tropical Medicine and Parasitology*, **54**, 132-140.

Juminer, B., Diallo, S. & Diagne, S. (1971) A focus of filariasis in Sandiara (Senegal). I. *Archives de l'Institut Pasteur de Tunis*, **48**, 231-246.

Knight, R. (1980) Current status of filarial infections in The Gambia. *Annals of Tropical Medicine and Parasitology*, **74**, 63-68.

Kuhlow, F. & Zielke, E. (1976) Distribution and prevalence of *Wuchereria bancrofti* in various parts of Liberia. *Tropenmedizin Und Parasitologie*, **27**, 93-100.

Lamontellerie, M. (1972) Results of surveys on the filariasis in western Upper Volta (cercle de banfora). *Annales de Parasitologie Humaine et Comparee*, **47**, 783-838.

Languillon, P. (1957) Carte des filaires du Cameroun. *Bulletin de la Société de Pathologie Exotique*, **3**, 417-427.

Massaga, J.J., Salum, F.M. & Savael, Z.X. (2000) Clinical and parasitological aspects of Bancroftian filariasis in Hale, northeast Tanzania. *Central African Journal of Medicine*, **46**, 237-241.

McConnell, E. & Dennis, D.T. (1976) A survey for *Wuchereria bancrofti* in Ethiopia. *Ethiopian Medical Journal*, **14**, 31-36.

Meyrowitsch, D.W., Simonsen, P.E. & Magesa, S.M. (2004) A 26-year follow-up of Bancroftian filariasis in two communities in north-eastern Tanzania. *Annals of Tropical Medicine and Parasitology,* **98**, 155-169.

Meyrowitsch, D.W., Simonsen, P.E. & Makunde, W.H. (1996) Mass diethylcarbamazine chemotherapy for control of Bancroftian filariasis: Comparative efficacy of standard treatment and two semi-annual single-dose treatments. *Transactions of the Royal Society of Tropical Medicine and Hygiene*, **90**, 69-73.

Moyou-Somo, R., Antoine Ouambe, M., Fon, E. & Bema, J. (2003) Enquete sur la filariose lymphatique dans sept villages du district de sante de Bonassama dans l'estuarie du Wouri, province du Littoral, Cameroon. *Médecine Tropicale*, **63**, 583-586.

Mukoko, D.A., Pedersen, E.M., Masese, N.N., Estambale, B.B. & Ouma, J.H. (2004) Bancroftian filariasis in 12 villages in Kwale district, coast province, Kenya - variation in clinical and parasitological patterns. *Annals of Tropical Medicine and Parasitology*, **98**, 801-815.

Myung, K., Massougbodji, A., Ekoue, S., Atchade, P., Kiki-Fagla, V. & Klion, A.D. (1998) Lymphatic filariasis in a hyperendemic region: A ten-year, follow-up panel survey. American Journal of Tropical Medicine and Hygiene, **59**, 222-6

Ngwira, B.M., Tambala, P., Perez, A.M., Bowie, C. & Molyneux, D.H. (2007) The geographical distribution of lymphatic filariasis infection in Malawi. *Filaria Journal*, **6**, 12.

Ngwira, B.M., Jabu, C.H., Kanyongoloka, H., Mponda, M., Crampin, A.C., Branson, K., Alexander, N.D. & Fine, P.E. (2002) Lymphatic filariasis in the Karonga district of northern Malawi: A prevalence survey. *Annals of Tropical Medicine and Parasitology*, **96**, 137-144.

Njenga, S.M., Muita, M., Kirigi, G., Mbugua, J., Mitsui, Y., Fujimaki, Y. & Aoki, Y. (2000) Bancroftian filariasis in Kwale district, Kenya*. East African Medical Journal*, **77**, 245-249.

Onapa, A.W., Simonsen, P.E., Pedersen, E.M. & Okello, D.O. (2001) Lymphatic filariasis in Uganda: Baseline investigations in Lira, Soroti and Katakwi districts. *Transactions of the Royal Society of Tropical Medicine and Hygiene*, **95**, 161-167.

Onwuliri, C.O.E. & Anosike, K. (1989) Filariasis in some parts of Bauchi state of Nigeria. In: Epidemiology in Health Care Delivery , Proc. 4th African Reg. Conf. of the IEA, pp. 91-98, Harare, Zimbabwe.

Poindexter, H.A. (1950) Filariasis Bancrofti studies in Liberia. *American Journal of Tropical Medicine and Hygiene*, **30**, 519-523.

Rakotomalala, R., Comolet, T., Rajonson, R. & Rakoto, B. (1995) Prevalence of Bancroftian filariasis in the region of Sainte Marie: Implications in public health. *Archives de l'Institut Pasteur de Madagascar*, **62**, 124-127.

Roberts, C.J., Whitehall, J. & Gelfand, M. (1973) W. Bancrofti in the Kanyemba area. *Central African Journal of Medicine*, **19**, 13-14.

Sasa, M. (1976) Human filariasis. A global survey of epidemiology and control. University Park Press: Baltimore.

Satti, M.H. & Abdel Nur, O.M. (1974) Bancroftian filariasis in the Sudan. *Bulletin of the World Health Organization*, **51**, 314-315.

Scheiber, P. & Braun-Munzinger, R.A. (1976) Bancroftian filariasis in Togo 1. A comparative field study of the membrane filtration concentration technique and conventional blood films. *Tropenmedizin Und Parasitologie*, **27**, 224-228.

Shawarby, A.A., Mahdi, A.H., Naguib, K. & Moharran, A. (1965) Incidence of filariasis in Egypt. *Journal of the Egyptian Public Health Association*, **40**, 267-282.

Simonsen, P.E., Meyrowitsch, D.W., Makunde, W.H. & Magnussen, P. (1995) Selective diethylcarbamazine chemotherapy for control of Bancroftian filariasis in two communities of Tanzania: compared efficacy of a standard dose treatment and two semi-annual single dose treatments. *American Journal of Tropical Medicine and Hygiene*, **53**, 267-272.

Simonsen, P.E., Meyrowitsch, D.W., Jaoko, W.G., Malecela, M.N., Mukoko, D., Pedersen, E.M., Ouma, J.H., Rwegoshora, R.T., Masese, N., Magnussen, P., Estambale, B.B. & Michael, E. (2002) Bancroftian filariasis infection, disease, and specific antibody response patterns in a high and a low endemicity community in East Africa. *American Journal of Tropical Medicine and Hygiene*, **66**, 550-559.

Southgate, B.A. (1992) Intensity and efficiency of transmission and the development of microfilaraemia and disease: their relationship in lymphatic filariasis. *Journal of Tropical Medicine and Hygiene*, **95**, 1-12.

Terranella, A., Eigiege, A., Gontor, I., Dagwa, P., Damishi, S., Miri, E., Blackburn, B., Mcfarland, D., Zingeser, J., Jinadu, M.Y. & Richards, F.O. (2006) Urban lymphatic filariasis in central Nigeria. *Annals of Tropical Medicine and Parasitology*, **100**, 163-172.

Udonsi, J.K. (1986) The status of human filariasis in relation to clinical signs in endemic areas of the Niger delta. *Annals of Tropical Medicine and Parasitology*, **80**, 425-432.

Ufomadu, G.O., Nwoke, B.E., Akoh, J.I., Sato, Y., Ekejindu, G.O., Uchida, A., Shiwaku, K., Tumbau, M. & Ugomo, K.K. (1990) The occurrence of loiasis, mansonellosis and wuchereriasis in the Jarawa river valley, central Nigeria. *Acta Tropica*, **48**, 137-147.

Wamae, C.N., Mwandawiro, C., Wambayi, E., Njenga, S. & Kiliku, F. (2001) Lymphatic filariasis in Kenya since 1910, and the prospects for its elimination: A review. *East African Medical Journal*, **78**, 595-603.

Wegesa, P., Mcmahon, J.E., Abaru, D.E., Hamilton, P.J., Marshall, T.F. & Vaughan, J.P. (1979) Tanzania filariasis project. Survey methodology and clinical manifestations of Bancroftian filariasis. *Acta Tropica*, **36**, 369-377.

Weil, G.J., Ramzy, R.M., El Setouhy, M., Kandil, A.M., Ahmed, E.S. & Faris, R. (1999) A longitudinal study of Bancroftian filariasis in the Nile delta of Egypt: baseline data and one-year follow-up. *American Journal of Tropical Medicine and Hygiene*, **61**, 53-58.

Wijeyaratne, P.M., Singha, P., Verma, O.P. & Motha, B. (1982) Evaluation of the diethylcarbamazine provocative test in the diagnosis of *Wuchereria bancrofti* infections in the Nigerian savanna and the effects on *Dipetalonema perstans*. *Transactions of the Royal Society of Tropical Medicine and Hygiene*, 76, 387-389.
