# Supplementary material for: Predicting the Current and Future Potential Distributions of Lymphatic Filariasis in Africa Using Maximum Entropy Ecological Niche Modelling
Source: PLoS One. 2012 Feb 16;7(2):e32202. doi: 10.1371/journal.pone.0032202 (PMC3281123; doi:10.1371/journal.pone.0032202)
Supplement: Appendix S2 — Comparison of GARP and Maxent model fits. Partial AUC ratios taken across 200 bootstrap replications are shown for omission errors, E, of 1 and 5. (DOCX) [file pone.0032202.s002.docx]

**Appendix S2:**

Comparison of GARP and Maxent model fits. Partial AUC ratios taken across 200 bootstrap replications are shown for omission errors, E, of 10 and 100.

|  |  | GARP |  |  | Maxent |
| --- | --- | --- | --- | --- | --- |
|  | E=10 | E=100 |  | E=10 | E=100 |
| Minimum | 1.4167 | 1.5180 |  | 1.3331 | 1.6934 |
| Maximum | 1.4167 | 1.5628 |  | 1.5927 | 1.8336 |
| Mean | 1.4167 | 1.5437 |  | **1.4656** | **1.7622** |
| Standard deviation | 0.0000 | 0.0075 |  | 0.0655 | 0.0270 |
| P-value | <0.00001 | <0.00001 |  | <0.00001 | <0.00001 |
